# High Expression of Lysophosphatidic Acid Induces Nerve Injury in LSS Patients via AKT Mediated NF-κB p65 Pathway

**DOI:** 10.3389/fphar.2021.641435

**Published:** 2021-03-18

**Authors:** Guiliang Zhai, Wenfei Liang, Yongjun Xu

**Affiliations:** ^1^ Orthopedic Surgery, Binzhou Central Hospital of Shandong Province, Binzhou, China; ^2^ Department of Stomatology, Binzhou Central Hospital of Shandong Province, Binzhou, ,China; ^3^ Xianyang Central Hospital of Shaanxi Province, Xianyang, China

**Keywords:** lumbar spinal stenosis, lysophosphatidic acid, Akt, NF-κB pathway 3, LSS

## Abstract

Lumbar spinal stenosis (LSS) is a spinal degenerative disease, complicated with nerve injury. Lysophosphatidic acid (LPA), a kind of glycerophospholipid molecule is elevated in the initial stages of neural injury. This research aimed to investigate the patho-mechanism of nerve injury caused by LPA in LSS patients. Twenty-five LSS patients and fifteen idiopathic scoliosis patients (without neurological symptoms) were recruited from Xianyang Central Hospital of Shanxi Province. We measured the concentration of LPA in cerebrospinal fluid samples of all subjects. Different concentrations (0.1, 1, and 10 mol/L) of LPA were used to stimulate Rat Neurons-spinal cord (RN-SC) cells. The effects of LPA on cell injury was detected by MTT and LDH (lactate dehydrogenase) assay. Cell apoptosis was determined by FCM (flow cytometry) and TUNEL staining. The changes in the expression of key proteins involved in Akt mediated NF-κB p65 pathway intervened by LPA were determined by western blot. RN-SC cells were pretreated with JSH-23 (NF-κB inhibitor) before LPA exposure, followed by cell apoptosis measurement. The concentration of LPA in LSS patients was notably higher than that in control patients (*p* < 0.01). The level of LPA was positively correlated with the severity of LSS. LPA treatment induced RN-SC cells displaying oval or rounded cell body with degenerated protrusion dose dependently. In addition, LPA decreased RN-SC cell viability and promoted cell apoptosis in a dose-dependent manner. LPA initiated Akt phosphorylation, IKB phosphorylation, and NF-κB nuclear translocation in a dose-dependent manner. However, JSH-23 (NF-κB inhibitor) pre-treatment prevented effects of LPA. The high levels of LPA induced nerve injury by reducing the viability of RN-SC cells and promoted cell apoptosis through Akt mediated NF-κB p65 signaling pathway. LPA might be a new therapeutic target for relieving nerve injury in LSS patients.

## Introduction

Lumbar spinal stenosis (LSS) is a spinal degenerative disease that has a high incidence in individuals between 50 and 79 years of age. LSS is attributed to the compression of cauda equina fibers and sensitization of the central and peripheral nervous systems. Nerve injury (at the peripheral and central nervous system) can lead to abnormal or overactive activity in regions of damaged nervous tissue, which is associated with severe and intractable neuropathic pain, tingling, numbness, tendon reflex loss, neurogenic claudication, and fatigue (Bouyer-Ferullo, 2013; Gibson et al., 2017; Jensen et al., 2020). The widespread and severe debilitating neuropathic pain has affected millions of people worldwide (Lee et al., 2019). The symptoms of LSS are aggravated by activities such as walking and standing. LSS seriously restricts the daily activities and affects life quality of patients. Current management strategies for LSS include surgery and nonoperative treatment (such as exercise, traction, epidural steroid injection) (Miyamoto et al., 2008). However, the therapeutic options were limited for poor prognosis. Therefore, the effective prevention of nerve injury in LSS patients in a long follow-up needs to be solved urgently.

Lysophosphatidic acid (LPA) is present in body fluids like serum and cerebrospinal fluid (CSF), which can be secreted by activated platelets. LPA exerts biological function in mediating wound healing, differentiation, survival and apoptosis (Yung et al., 2014). LPA as a novel survival/apoptosis factor, mediates the survival of Schwann and T lymphoma cells (Weiner and Chun, 1999; Gupta et al., 2020). Conversely, the researchers indicate that LPA is significantly increased in the initial stages of neural injury (Mihara et al., 2019). Kuwajima et al. indicated that LPA was associated with the pain intensity and symptom in patients with neuropathic pain (Kuwajima et al., 2018). LPA is responsible for the nerve damage and neuropathic pain in osteoarthritis (Mcdougall et al., 2017). LPA triggers neuronal cell death in brain after ischemic injury (Wang et al., 2018). Recently, it has been demonstrated that the level of LPA in the CSF of LSS patients was significantly increased (Theus et al., 2017; Hayakawa et al., 2019). However, the role of LPA in nerve damage of LSS patients has not been clarified.

NF-κB transcription family factors play an important part in the regulation of gene expression for stress-associated, inflammatory, and cell survival. It has been reported that LPA can regulate cell survival through Akt/NF-κB signaling pathway (Raj et al., 2004; Hwang et al., 2016). Mounting evidence indicated that the activation of NF-κB signaling pathway was relevant to nerve injury (Theus et al., 2017; Mettang et al., 2018). Indeed, it has also been demonstrated that Akt signaling pathway is involved in neuronal injury (Zhou et al., 2019; Wang et al., 2020). Phosphorylation of Akt can activate IKKs, leading to phosphorylation and degradation of IKB, p65 nuclear translocation (Ya et al., 2018). Whether Akt mediated NF-κB signaling is involved in LPA caused nerve injury in LSS patients is still unclear.

In this study, we used Rat Neurons-spinal cord (RN-SC) to examine the association between LPA and neural damage, and investigate whether LPA was involved in nerve injury by activating Akt mediated NF-κB p65 signaling pathway. We aimed to provide a new perspective for the treatment of nerve injury for LSS patients.

## Materials and Methods

### Ethical Approval and Patients and LPA Concentration Determination

Twenty-five LSS patients (13 males and 12 females, mean age: 68 ± 8 years) and fifteen idiopathic scoliosis patients (without neurological symptoms, seven males and eight females, mean age: 77 ± 6 years) were included from Xianyang Central Hospital of Shanxi Province between March 2017 and November 2019. The study was approved by Xianyang Central Hospital Ethical Committee on 11–12-2017. The diagnosis of LSS was based on physician assessment of appropriate symptoms, and imaging examination. All the subjects completed Neuropathic Pain Symptom Inventory (NPSI) questionnaire (Bouhassira et al., 2004) to assess the pain symptom. CSF samples were obtained from all subjects by lumbar puncture, and the LPA concentration in CSF was measured by ELISA kit (#T-2800S, Echelon Bioscience, United States) in accordance with the previous description (Gotoh et al., 2019).

### Cell Culture

RN-SC cells were purchased from Chinese Ningbo Mingzhou Biological Technology Co. LTD. (Zhejiang, China). RN-SC cells were cultured in Neurobasal-A medium (Gibco, Waltham, MA, United States United States) containing 1% penicillin-streptomycin mixed solution in a 5% CO_2_ humidified incubator at 37°C as previously described (He et al., 2018).

### MTT Assay and LDH Assay

RN-SC cells (5 × 10^3^ cells/well) were plated in 96-well plates for 24 h. LPA (Sigma, Olney, MD, United States) was dissolved in 1% DMSO and diluted with PBS at a series of concentrations (0, 0.1, 1, and 10 μmol/L). RN-SC cells were stimulated with different concentrations of LPA for 24 h (Zhang et al., 2020), and the cells stimulated with LPA (0 μmol/L) were considered as the vehicle control. For MTT assay, the medium was added with 10 μL MTT solution (Beyotime, Shanghai, China) for 4 h, then 100 μL DMSO was applied to wells for 10 min. The optical density (OD) at 570 nm was detected.

The culture medium of each well was collected for LDH determination using LDH cytotoxicity assay kit (Beyotime, Shanghai, China). The OD at 490 nm was measured by microplate reader (Thermo, Carlsbad, United States). Each experiment was carried out in triplicate.

### Cell Morphology

Following treatment, the changes of cell morphology and axons were photographed under fluorescence microscope (Leica, Solms, Germany).

### TUNEL Assay

LPA induced apoptosis in cultured neurons was tested by TUNEL assay (He et al., 2018). Approximately 5 × 10^5^ cells/well RN-SC cells were plated in 6-well plates for 24 h. Briefly, cells from the different treatment groups were fixed, and permeabilized. Then 20 μL TUNEL reaction solution (Beyotime, Shanghai, China) was added into wells at 37°C for 1 h. After that, cells were treated with DAPI (10 μg/ml, Beyotime, Shanghai, China) for 10 min. The apoptosis RN-SC cells were detected by fluorescence microscope (Leica, Solms, Germany). TUNEL-positive staining represented apoptotic cells.

### Flow Cytometry Analysis

RN-SC cell apoptosis induced by LPA was also detected by FCM (Behbahani et al., 2005). Cells treated with LPA were washed and harvested, then neuronal cultures were treated with EDTA and 0.05% trypsin. Cells were washed with PBS and collected by centrifugation at 1,500 rpm. Subsequently, cultures were resuspended in annexin-binding buffer and incubated with PI buffer (Thermo Fisher, Massachusetts, America) and FITC-Annexin V (Thermo Fisher, Massachusetts, America) for 15 min in dark at 37°C. The cell apoptotic rate of RN-SC was monitored using the FCM (BD, Franklin Lakes, NJ, United States).

### Western Blotting

The levels of apoptosis-related proteins (caspase-3, Cleaved caspase-3, Bax) and pathway related proteins (Akt, Phospho-Akt, IKBα, Phospho-IKBα and p65) in RN-SC cells was detected by western blotting (Schichl et al., 2009; Zhao et al., 2018). For nucleoprotein extraction, cells were resuspended in cell lysis buffer supplemented with 10% PMSF on ice. After centrifugation, the precipitation was resuspended in the nuclei lysis buffer (Beyotime, Shanghai, China) to obtain the nucleoprotein for detecting p65 expression. The cells were lyzed in 400 μl RIPA buffer (Thermo Fisher Scientific, Carlsbad, United States) to extract the total proteins. The protein concentration was determined by BCA kit (Beyotime, Shanghai, China). Total proteins (20 μg) were fractionated by SDS-PAGE and transferred onto PVDF (Bio-Rad, Hercules, CA, United States) membranes, which were sequentially blocked with 5% skim milk. The membranes were sequentially incubated with primary antibodies [caspase-3 (1:1000, #9662, CST, Boston, America), Cleaved caspase-3 (1:1000, #9664, CST, Littleton, CO, United States), Bax (1:1000, #2772, CST, Littleton, CO, United States), Akt (1:1000, #4691, CST, Littleton, CO, United States), Phospho-Akt (1:1000, #4060, CST, Littleton, CO, United States), IKBα (1:1000, #4812, CST, Littleton, CO, United States), Phospho-IKBα (1:1000, #2859, CST, Littleton, CO, United States), p65 (1:1000, #8242, CST, Littleton, CO, United States), *β*-actin (1:1000, #8457, CST, Littleton, CO, United States), Bcl-2 (1:1000, #33-6100, Thermo Fisher Scientific, Carlsbad, United States)] at 4°C overnight. Then the membranes were incubated with peroxidase-conjugated secondary antibody anti-rabbit IgG (1:2000, #7074, CST, Littleton, CO, United States) or anti-mouse IgG (1:2000, #4410, CST, Littleton, CO, United States) for 2 h at room temperature (RT). The immunoreactive bands were visualized with an enhanced chemiluminescence reagent kit (32109, Thermo Fisher Scientific, Carlsbad, United States). The intensities of protein bands were quantitatively analyzed by the ImageJ software (Bethesda, MD, United States) and the relative expression of target protein was normalized with *β*-actin.

### Immunohistochemistry

Nuclear translocation of p65 induced by LPA was observed by immunohistochemistry. Briefly, cells were fixed and permeabilized, then incubated with blocking buffer for 1 h. Samples were incubated with primary antibodies (p65, 1:400, #8242, CST, Littleton, CO, United States) overnight at 4°C and secondary antibodies (1:1000, #2985, CST, Littleton, CO, United States) for 1 h at RT. The images were obtained by fluorescence microscope (Leica, Solms, Germany).

### Statistical Analysis

The data were present as the mean ± standard deviation (SD) and analyzed by SPSS 16.0. Statistical comparisons were made using the Student’s t-test or one-way ANOVA (Analysis of Variance) with Tukey's test. When *p* < 0.05, *p* < 0.01 and *p* < 0.001, significant differences were considered.

## Results

### The High Expression of LPA in CSF of LSS Patients

According to the median value of NPSI score, LSS patients were divided into two groups, including mild group (n = 12) and severe group (n = 13). The NPSI score of patients in control group were 0. The results revealed that the levels of LPA in LSS patients was significantly higher than controls (*p* < 0.001), and the levels of LPA in severe patients was significantly higher compared with mild patients (Table 1, *p* < 0.001). The evidences revealed that LPA was significantly accumulated in the CSF of LSS patients, and the level of LPA was positively correlated with the severity of LSS.

**TABLE 1:** The levels of LPA in CSF between control group and LSS patient group.

		Control (n = 15)	LSS (n = 25)	*p* Value
Gender	Male (n = 13)	—	3.46 ± 0.31	0.259
	Female (n = 12)	—	3.29 ± 0.42	
LPA (μmol/L)		2.77 ± 0.09	3.36 ± 0.35	<0.001
	Mild (n = 12)		3.11 ± 0.20	<0.001
	Severe (n = 13)		3.63 ± 0.27	

LSS, lumbar spinal stenosis; LPA, lysophosphatidic acid LPA, CSF, cerebrospinal fluid. The inter-group and intro-group difference was analyzed by *t* test with the application of SPSS 16.0 software.

### The Optimum Concentration for LPA Applied to RN-SC Cells

**FIGURE 1:**
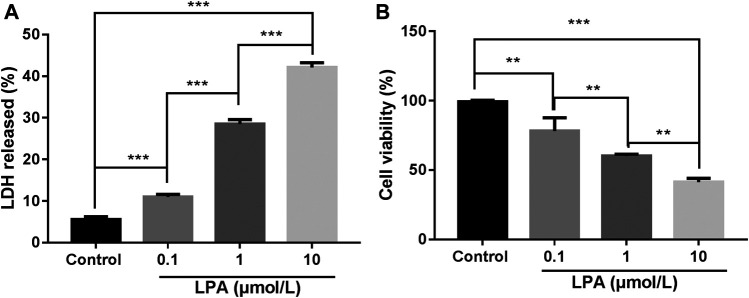
LPA reduced the viability of RN-SC cells. RN-SC cells (5 × 10^3^ cells/well) were seeded into 96-well plates and treated with LPA (0, 0.1, 1, and 10 μmol/L) for 24 h. **(A)** The LDH release was measured by LDH assay kit. **(B)** Cell viability was measured by MTT assays. All experiments were repeated for at least three times. Cells treated with 0 μmol/L LPA was considered as control. Data are expressed as mean ± SD. The differences among groups were analyzed by one-way ANOVA, followed by Tukey’s analysis. **p < 0.01, ***p < 0.001.

That LDH released into the cell culture medium indicates the damage of the cell membrane structure (Zhou et al., 2018) and MTT assay was used to measure the cell viability. To screen out the optimal concentration of LPA on RN-SC cells, cells were incubated with increasing doses of LPA from 0 to 10 μmol/L for 24 h. LPA dose dependently increased LDH release in RN-SC cells compared with the control group (*p* < 0.001, Figure 1A). MTT results also showed that cell viability of RN-SC induced by LPA, decreased dose dependently compared with control group (*p* < 0.01, Figure 1B). Combining LDH and MTT results, the optimal concentration of LPA was about 1 μmol/L, thus the concentration (1 μmol/L) was applied for relevant experiments.

### LPA Promotes RN-SC Cells Apoptosis

**FIGURE 2:**
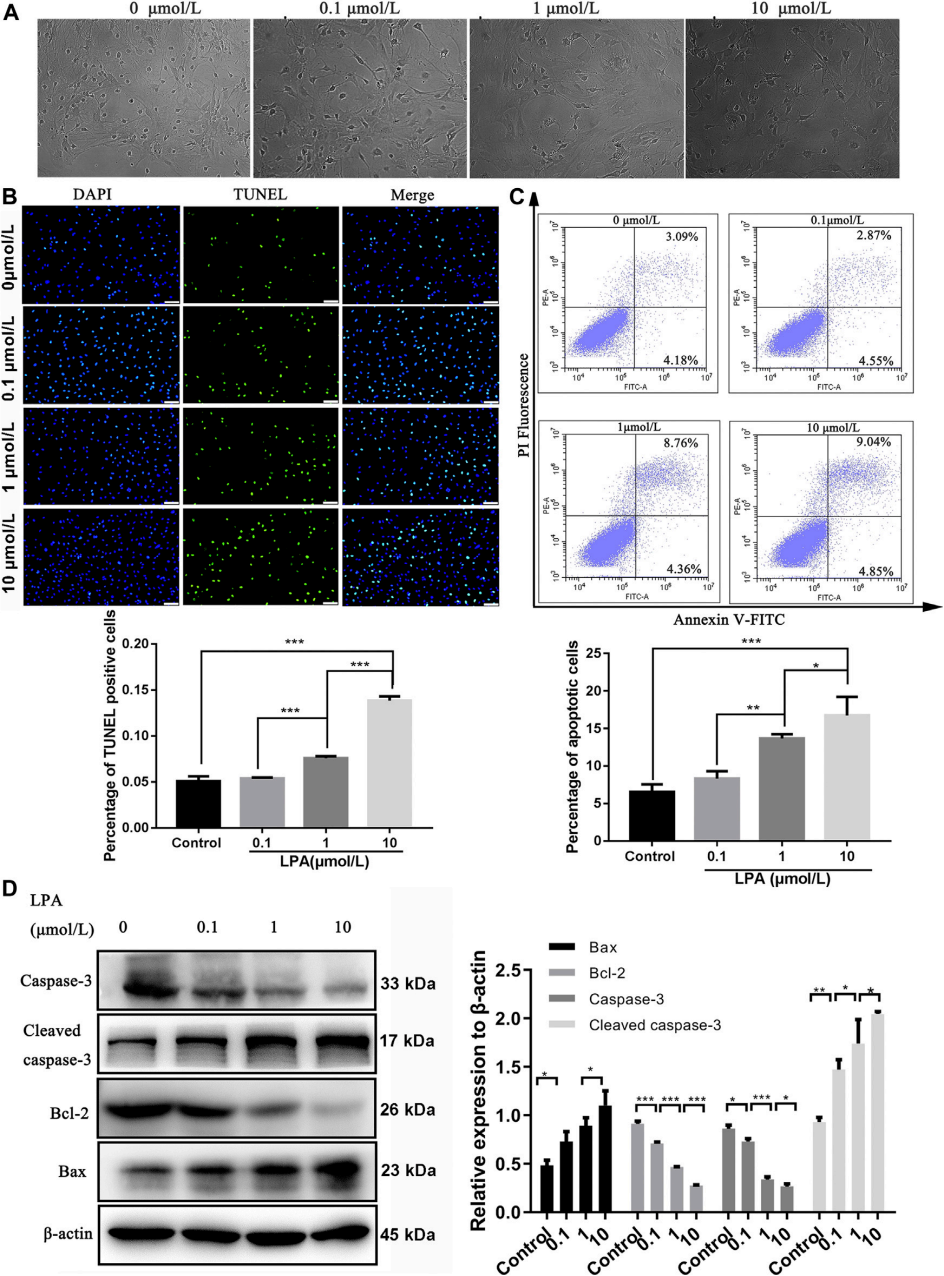
LPA promotes RN-SC cells apoptosis. RN-SC cells (5 × 10^3^ cells/well) were seeded into 96-wells plates and treated with LPA (0, 0.1, 1, and 10 μmol/L) for 24 h. **(A)**The morphological changes were observed by fluorescence microscope. **(B)** Cell apoptosis was analyzed by TUNEL staining. The percentage of TUNEL positive cells was calculated. **(C)** Cells were collected and double stained by Annexin-V and PI. The apoptotic ratio was detected by flow cytometry. **(D)** The expressions of apoptosis related proteins (caspase-3, Cleaved caspase-3, Bax and Bcl-2) were measured by western blot. Caspase-3 refers to pro-caspase-3 in this study. The Data are representative of three independent experiments. Data are expressed as mean ± SD and the multi-group comparison was performed by one-way ANOVA. *p < 0.05, **p < 0.01, ***p < 0.001.

As illustrated in Figure 2A, LPA induced morphological changes of RN-SC cells displaying oval or rounded cell body with degenerated protrusion in a dose dependent manner (Figure 2A). The TUNEL assay results showed the percentage of TUNEL positive cells was significantly increased dose dependently after LPA exposure compared with the control group (*p* < 0.001, Figure 2B). The same trend was observed in flow cytometry results, the apoptotic ratio was 6.53 ± 0.84% in control group, whereas LPA exposure (1, 10 μmol/L) increased the apoptotic ratio (13.68 ± 0.47%, 16.75 ± 2.03%) respectively. Results revealed that treatment with LPA markedly increased the apoptotic ratio of RN-SC cells in a dose dependent manner (*p* < 0.05, Figure 2C).

The changed expression of apoptosis-related proteins induced by LPA were assessed by western blot. The results revealed that the levels of Bcl-2 and caspase-3 significantly downregulated with increasing dose of LPA (*p* < 0.05). Conversely, the expression levels of Bax and cleaved caspase-3 were significantly elevated by LPA dose dependently (*p* < 0.05, Figure 2D). These evidences clearly indicated that LPA induced RN-SC cells apoptosis dose dependently.

### LPA Activated NF-κB p65 Signaling Through Akt to Mediate RN-SC Cell Injury

**FIGURE 3:**
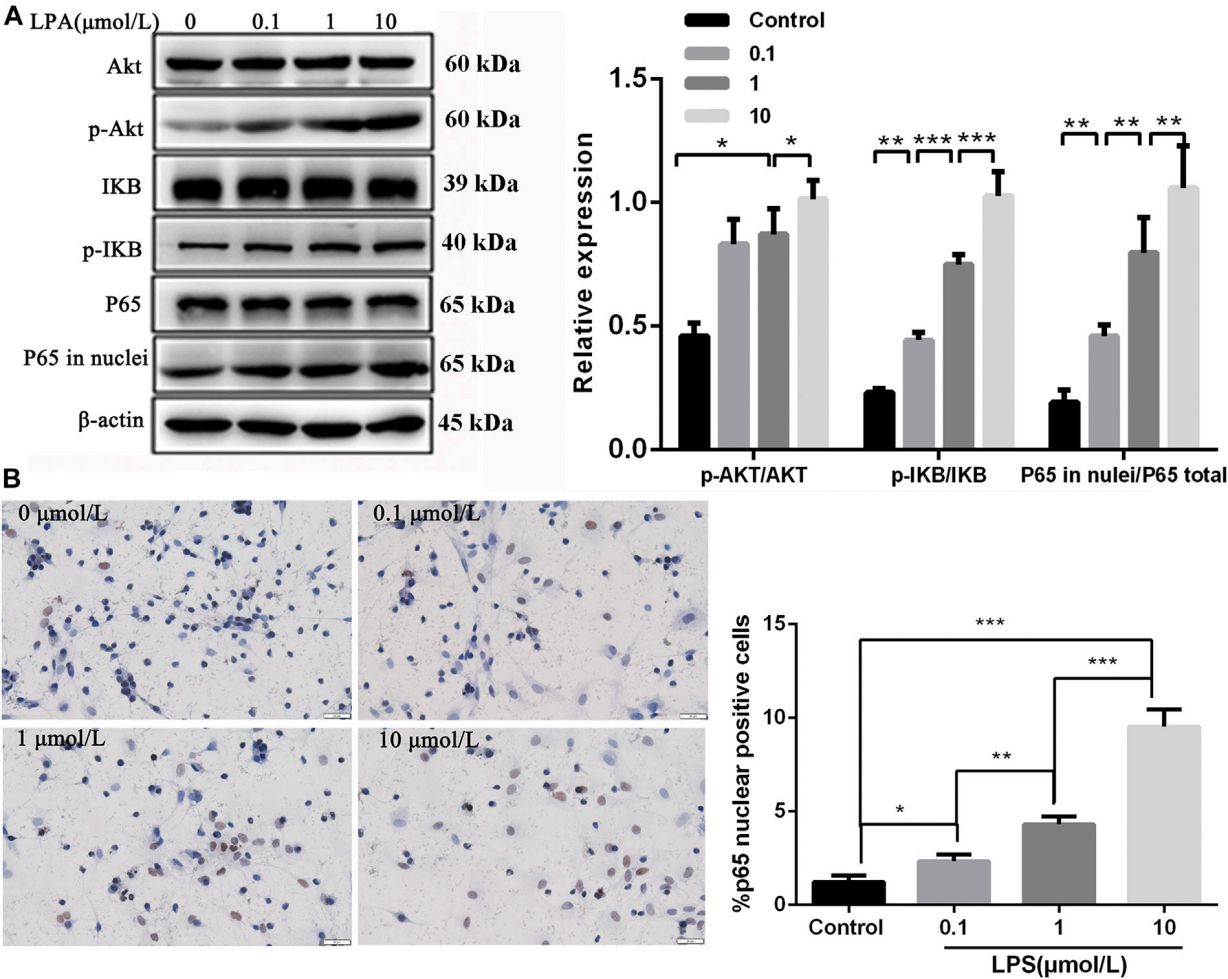
Akt mediated NF-κB p65 signaling was activated in RN-SC cells mediated by LPA. RN-SC cells (5 × 10^5^ cells/well) were seeded into 6-wells plates and treated with LPA (0, 0.1, 1, and 10 μmol/L) for 24 h. **(A)** The expressions of AKT, *p*-AKT, IKB, *p*-IKB, total p65 and p65 in nuclei were measured by western blot. **(B)** The p65 translocation was detected by immunohistochemistry**.** Data are representative of three independent experiments. Data are expressed as mean ± SD. RN-SC cells treated with 0 μmol/L LPA were considered as controls. The differences of protein expression and P65 translocation were analyzed by one-way ANOVA, followed by Tukey's method. *p < 0.05, **p < 0.01, ***p < 0.001.

To explore whether LPA induced RN-SC cells injury via activating NF-κB p65 signaling through Akt, cells were treated with LPA at different concentrations (0, 0.1, 1, and 10 μmol/L), followed by key protein detection in Akt mediated NF-κB p65 signaling. Western blot showed that LPA treatment increased phosphorylation of Akt dose dependently compared with control group (*p* < 0.05) (Figure 3A). The level of phosphorylated IKB was elevated, and the level of p65 in nuclei was obviously increased following LPA exposure dose-dependently (all *p* < 0.01, Figure 3A). Immunohistochemistry results also showed p65 protein was significantly accumulated in nuclei of RN-SC cells after LPA exposure in a dose-dependent manner (*p* < 0.05, Figure 3B). These evidences verified that LPA activated NF-κB p65 signaling through Akt dose dependently in RN-SC cells.

**FIGURE 4:**
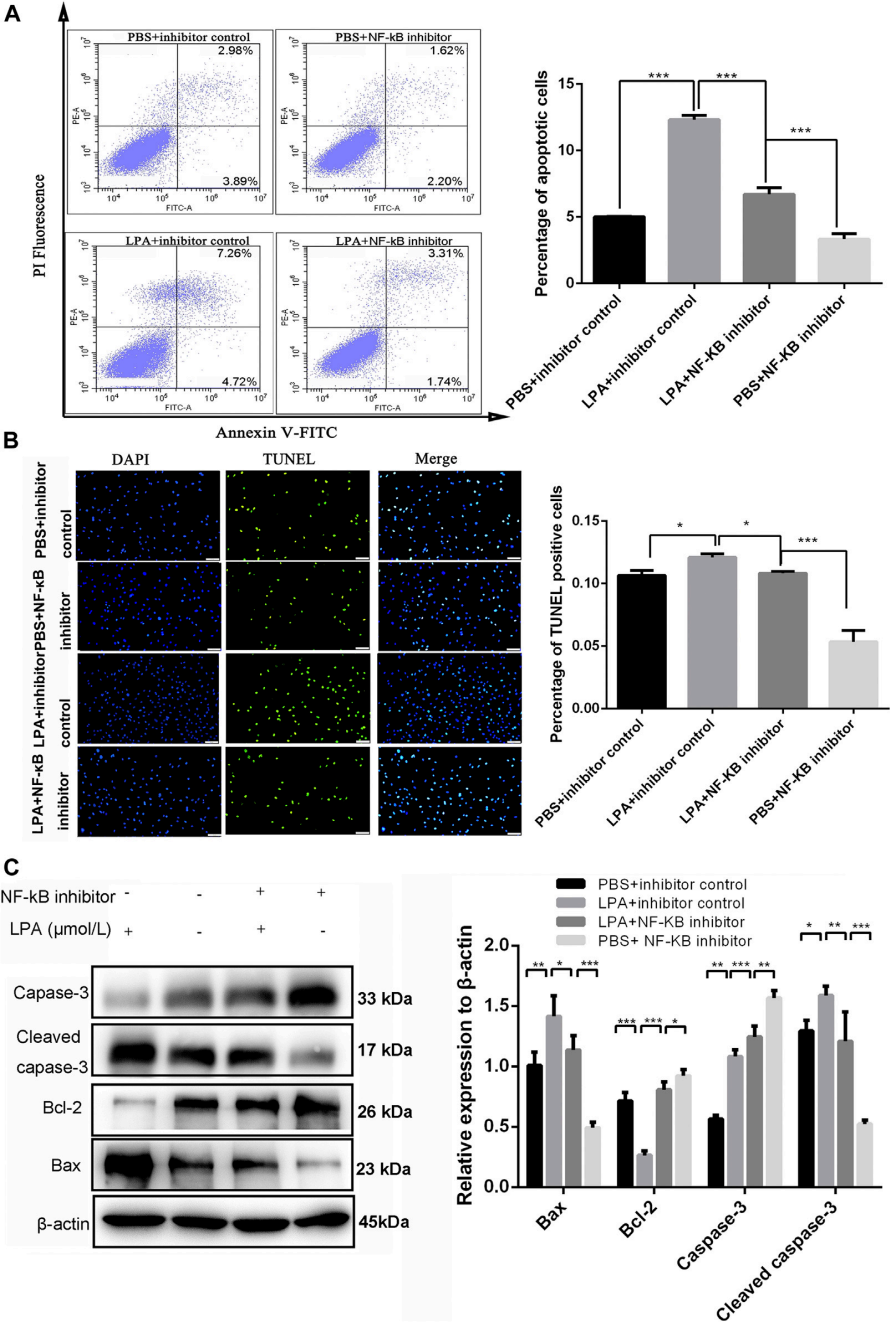
LPA induced RN-SC cells apoptosis by NF-κB p65 pathway. RN-SC cells (5 × 10^5^ cells/well) were seeded into 6-wells plates and treated with NF-κB inhibitor (JSH-23, 10 μM) before LPA (1 μmol/L) exposure. **(A)** The apoptotic ratio was measured by flow cytometry. **(B)** The percentage of TUNEL positive cells was measured by TUNEL staining. **(C)** The expressions of caspase-3, cleaved caspase-3, Bax and Bcl-2 were measured by western blot**.** Caspase-3 refers to pro-caspase-3 in this study. RN-SC cells treated with 0 μmol/L LPA were considered as controls. Data are representative of three independent experiments. Data are expressed as mean ± SD. The inter-group differences were analyzed by one-way ANOVA with Tukey's test. *p < 0.05, **p < 0.01, ***p < 0.001.

To investigate whether NF-κB p65 signaling was involved in RN-SC cell injury induced by LPA, cells were pre-treated with JSH-23 (10 μM) for 24 h before LPA (1 μmol/L) exposure. The flow cytometer results showed that the apoptotic rate of RN-SC cells was 6.71 ± 0.41% in the PBS + inhibitor control group, whereas LPA exposure markedly increased the RN-SC cells apoptotic ratio (12.3 ± 0.27%) in the LPA + inhibitor control group. However, JSH-23 remarkably reduced the cell apoptotic rate (5.01 ± 0.03%) on LPA induced RN-SC cells in LPA + NF-κB inhibitor group (*p* < 0.001, Figure 4A). The similar results were observed in TUNEL staining. The percentage of TUNEL positive cells in LPA + inhibitor control group was significantly higher than that in PBS + inhibitor control group, while JSH-23 pre-treatment relieved cell apoptosis induced by LPA (Figure 4B). Meanwhile, JSH-23 pre-treatment abolished the changes in the expressions of apoptosis related proteins induced by LPA (Figure 4C). All these indicated that LPA induced RN-SC cell injury through Akt mediated NF-κB p65 signaling.

## Discussion

LPA is increasedly secreted from platelets following neural injury, but little is known about the role of LPA in LSS. In the current study, the concentration of LPA in LSS patients was measured and the effect of LPA on neural injury were detected. The signaling pathway involved in neural injury induced by LPA was explored. Our data showed that LPA induced the reduction of RN-SC cell viability, the elevation of LDH release and cell apoptosis dose dependently. NF-κB p65 pathway mediated by Akt was activated in LPA induced nerve cell injury.

K. Hayakawa et al. indicated that the concentration of LPA in the LSS patients was significantly higher than that in controls (Hayakawa et al., 2019). In the present study, we also measured the level of LPA in the CSF of LSS patients with nerve injury and idiopathic scoliosis patients (without neurological symptoms). Our results determined that the level of LPA was elevated in the CSF of LSS patients, and the expression level of LPA was positively correlated with the severity of LSS, which was consistent with the previous study mentioned above. A line of evidences showed that LPA could induce neuronal apoptosis *in vitro* (Moolenaar et al., 1997; Steiner et al., 2000). Moolenaar et al. found that LPA directly caused neuronal degeneration under pathological conditions (Moolenaar et al., 1997). Steiner et al. reported that LPA could significantly induce the apoptosis of hippocampal neurons and neuronal PC12 cells (Steiner et al., 2000). In this study, we added the exogenous LPA into cultured RN-SC cells, and identified that LPA induced RN-SC cell apoptosis, which was in agreement with the previous findings (Steiner et al., 2000). In addition, our data showed that the LPA exposure caused axons degeneration of RN-SC cells, increased cell volume and promoted cell rounding. LDH and MTT assay proved that LPA significantly reduced cell viability. Flow cytometry and TUNEL staining also demonstrated that LPA induced RN-SC cell apoptosis in a dose dependent manner. It is reported that the overexpression of Bcl-2 presents neuroprotective effect against nerve cell apoptosis in rats following cerebral ischemia. Zhang et al. found that LPA induced PC12 cells apoptosis, reflected by declined Bcl-2 expression and increased expression of Bax (Zhang et al., 2020). In this study, we also found that LPA increased the expression levels of apoptosis related factors (Bax, Cleaved caspase-3) and reduced Bcl-2 and pro-caspase activity. Our results were in agreement with previous results (Zhang et al., 2015; Zhang et al., 2020), and reminded that the high levels of LPA were relevant to nerve injury in LSS patients.

Akt, is a serine/threonine protein kinase, whose phosphorylation is related with neuronal proliferation and differentiation (Song et al., 2018). It is reported that AKT/p38/ERK/NFκB-mediated pathway was activated in reduced endothelial cell proliferation by androgen receptor (Huo et al., 2019). Wang et al. suggested that Homer1a relieved neuronal injury induced by traumatic damage by inhibiting PI3K/AKT/mTOR signaling (Wang et al., 2020). PI3K/Akt/mTOR signaling was also involved in oxygen-glucose deprivation/re-oxygenation-induced neuron injury (Zhou et al., 2019). In addition, NF-κB pathway can be activated by Akt protein kinases and involved in various functions, such as immune, inflammation, apoptosis. As described in the previous study, Akt/NF-κB signaling was involved in reduced proliferation, metastasis and increased apoptosis of glioma cells induced by eriodictyol (Song et al., 2018). Akt/NF-κB pathway was activated in the LPA-induced hypertrophy of cardiac myocytes (Chen et al., 2008). Besides, NF-κB served as a p50/p65 heterodimer, tightly binds to IKB, a NF-κB inhibitory protein. In stimulated cells, IKB was phosphorylated and degraded and p50/p65 heterodimer was released from the IKB complex to translocate to the nucleus and regulate apoptosis related gene expression (Fan et al., 2004). In the current study, we found that Akt phosphorylation, IKB phosphorylation and p65 nuclear translocation significantly increased in RN-SC cells after LPA treatment, accompanied with increased cell apoptosis. The rescued experiment showed that JSH-23 (NF-κB inhibitor) pre-treatment partly abolished the effect of LPA on RN-SC cells. All these suggested that LPA could regulate RN-SC cell apoptosis through Akt mediated NF-κB p65 signaling pathway.

## Conclusion

In summary, LPA level was significantly elevated in LSS patients and correlated with the severity of LSS. LPA exposure promoted neuronal apoptosis and morphologic changes by activating NF-κB p65 pathway through Akt. Therefore, LPA might be a biomarker for nerve injury and a therapeutic target for nerve injury in LSS patients.

## Data Availability

The raw data supporting the conclusions of this article will be made available by the authors, without undue reservation.
